# Uptake of meningococcal conjugate vaccine among adolescents in large managed care organizations, United States, 2005: Demand, supply and seasonality

**DOI:** 10.1186/1471-2334-9-175

**Published:** 2009-11-03

**Authors:** Suchita A Lorick, Daniel Fishbein, Eric Weintraub, Pascale M Wortley, Grace M Lee, Fangjun Zhou, Robert Davis

**Affiliations:** 1 Immunization Services Division, National Center for Immunization and Respiratory Diseases, Centers for Disease Control & Prevention, 1600 Clifton Road, MS E-52, Atlanta, Georgia, 30333, USA; 2 Epidemic Intelligence Service, Office of Workforce and Career Development, Centers for Disease Control and Prevention, 1600 Clifton Road, MS E-52, Atlanta, GA 30333, USA; 3 Division of Global Migration and Quarantine, National Center for Preparedness, Detection, and Control of Infectious Diseases, Center for Disease Control and Prevention, 1600 Clifton Road, MS E-03, Atlanta, GA 30333, USA; 4 Immunization Safety Office, Centers for Disease Control and Prevention, 1600 Clifton Road, MS D-26, Atlanta, GA 30333, USA; 5 Department of Ambulatory Care & Prevention, Harvard Medical School & Harvard Pilgrim Health Care, Department of Population Medicine, 133 Brookline Avenue, 6th floor, Boston, MA 02215, USA; 6 Center for Health Research, Southeast, Kaiser Permanente, Center for Health Research, Southeast, 10 Piedmont Center, 3495 Piedmont Road NE, Suite 205, Atlanta, GA 30305, USA

## Abstract

**Background:**

In February 2005, the US Advisory Committee on Immunization Practices recommended the new meningococcal conjugate vaccine (MCV4) for routine use among 11- to 12-year-olds (at the preadolescent health-care visit), 14- to 15-year-olds (before high-school entry), and groups at increased risk. Vaccine distribution started in March; however, in July, the manufacturer reported inability to meet demand and widespread MCV4 shortages were reported. Our objectives were to determine early uptake patterns among target (11-12 and 14-15 year olds) and non-target (13- plus 16-year-olds) age groups. A post hoc analysis was conducted to compare seasonal uptake patterns of MCV4 with polysaccharide meningococcal (MPSV4) and tetanus diphtheria (Td) vaccines.

**Methods:**

We analyzed data for adolescents 11-16 years from five managed care organizations participating in the Vaccine Safety Datalink (VSD). For MCV4, we estimated monthly and cumulative coverage during 2005 and calculated risk ratios. For MPSV4 and Td, we combined 2003 and 2004 data and compared their seasonal uptake patterns with MCV4.

**Results:**

Coverage for MCV4 during 2005 among the 623,889 11-16 years olds was 10%. Coverage for 11-12 and 14-15 year olds was 12% and 11%, respectively, compared with 8% for 13- plus 16-year-olds (*p *< 0.001). Of the 64,272 MCV4 doses administered from March-December 2005, 73% were administered June-August. Fifty-nine percent of all MPSV4 doses and 38% of all Td doses were administered during June-August.

**Conclusion:**

A surge in vaccine uptake between June and August was observed among adolescents for MCV4, MPSV4 and Td vaccines. The increase in summer-time vaccinations and vaccination of non-targeted adolescents coupled with supply limitations likely contributed to the reported shortages of MCV4 in 2005.

## Background

Menactra^®^, a quadrivalent meningococcal conjugate vaccine (MCV4) designed to prevent invasive disease due to *Neisseria meningitidis *was licensed in the United States in January 2005[1]. After consideration of disease epidemiology, anticipated supply and estimated demand, in February 2005, the Advisory Committee on Immunization Practices (ACIP) recommended MCV4 for adolescents ages 11 and 12 (at the preadolescent health-care visit) and, for those not previously vaccinated, before high-school entry (15 years old), and to persons at increased risk of invasive disease, including college freshmen living in dormitories[2]. This was the first *N. meningitidis *vaccine recommended for routine vaccination of adolescents in specific age groups. The recommendations also stated that other adolescents, college students, and persons infected with human immunodeficiency virus may elect to receive the vaccine. Previously, the ACIP had recommended that health care providers who care for college students (especially those living in dormitories) inform the students and their parents about meningococcal disease and the polysaccharide vaccine, Memomune^® ^(MPSV4)[3]. Additionally, ACIP had recommended that colleges inform students about the disease and the availability of MPSV4.

Distribution of MCV4 started in March, and ACIP recommendations became official on May 30, 2005 (upon publication in the Morbidity and Mortality Weekly Reports)[1]. Two months later, in July, the manufacturer reported inability to meet demand and placed limits on vaccine orders[4]. Additionally, on September 30, 2005, Centers for Disease Control and Prevention (CDC) and the Food and Drug Administration announced an investigation of a possible association between MCV4 and Guillain-Barré Syndrome (GBS)[5]. We analyzed data from 5 managed care organizations to study uptake patterns of MCV4 among adolescents during 2005 in relation to ACIP recommendations, and the timing of licensure through the GBS investigation[6]. Additionally, because we observed seasonal trends in MCV4 uptake, we sought to determine whether the trend was typical of meningococcal vaccines or other vaccines administered to adolescents. Therefore, we conducted a post hoc analysis of 2003-2004 VSD data to examine seasonal uptake of MPSV4 and tetanus diphtheria (Td) among adolescents. A booster dose of Td vaccine was recommended for adolescents at 11-12 or 14-16 years and every 10 years thereafter[7].

## Methods

### Vaccine Safety Datalink

The VSD is a collaborative project between CDC and eight US managed care organizations (MCOs) who have approximately six million members (2% of the US population)[6]. MCOs participating in VSD use an automated tracking system which records each vaccination administered to its members in addition to gathering data on member demographics and medical care utilization. The VSD's main purpose is to help detect possible vaccine related adverse events; however, VSD has also been utilized to study uptake of various vaccines [8-11].

### MCV4 Coverage

To examine MCV4 uptake during March-December 2005, we used 2005 vaccination data for adolescents 11-16 years of age from five of the eight MCOs (defined here as MCO A-E) who participated in ongoing data analyses for vaccine adverse events [10,12]. The institutional review board at each VSD site approved this study and agreed that informed consent from individuals was not required (Protocol #5126). We did not analyze data for 17 and 18 years olds because we could not determine which of the 17 and 18 year olds were college freshmen living in dormitories and therefore, recommended to receive MCV4. Additionally, in 2005, not all MCOs collected data for 18 year-olds.

Based on ACIP's recommendations for MCV4, adolescents 11-12 and 14-15 years of age were defined as "target" age groups. Although the ACIP recommendations specified 15 years of age to approximate high school entry, we combined 14 and 15 year olds to represent high school entry because census data estimate that 64% and 22% of 9^th ^graders are 14 and 15 years of age, respectively [1,13]. Adolescents aged 13 or 16 years were combined into the "13- plus 16-year-olds" year age group to represent the comparison or "non-target" age group.

We calculated the number of MCV4 doses administered by month and age group. To calculate MCV4 coverage, the number of vaccinated adolescents was divided by the total number of adolescents enrolled in the age group per month. The monthly enrollment for each MCO was based on 2005 data for all except MCO E; we used 2004 data for MCO E because 2005 data were not available at the time of this analysis. The yearly enrollment by age for each MCO was estimated by averaging the 12 monthly totals. We used SAS 9.1 to calculate chi-square p-values, risk ratios and 95% confidence intervals for 11-12 year olds and 14-15 year olds to receive MCV4 compared to 13- plus 16-year-olds.

Additionally, in February 2006, we queried each of the five MCO principal investigators about whether or not: 1) the MCO experienced a "vaccine shortage" during 2005 and 2) if they were aware of any local or state legislation that could have impacted MCV4 during 2005.

### Td and MPSV4 Uptake

We focused on the Td vaccine because it was the only vaccine recommended for adolescents during 2003-2004; unlike hepatitis B, measles mumps and rubella, and varicella vaccines, it is not recommended until age 11-12 and is not given in young children. For Td uptake, we combined preservative-free and non-preservative free tetanus and diphtheria toxoids, adsorbed, for adult use. To estimate MPSV4 and Td uptake, we combined 2003 and 2004 VSD data for adolescents 11-16 years of age from the same five MCOs (i.e A-E) used for examining MCV4 uptake. Two years of data were combined to maximize sample size and allow us to examine vaccination trends before MCV4 was licensed. Coverage for MPSV4 was not calculated since this vaccine was not routinely recommended for all 11-16 year old adolescents and eligibility (i.e. denominator) could not be accurately ascertained in this study. Similarly, Td coverage was not estimated because the indications for Td use (e.g. for use after tetanus-prone injury) were not known. 2005 data were not analyzed since the introduction of MCV4 likely affected the uptake patterns of MPSV4 (i.e. MCV4 substituted for MPSV4) and Td (e.g. uptake increased perhaps due to more immunization visits related to introduction of MCV4).

Therefore, for both MPSV4 and Td, we calculated the number of doses administered by month. MPSV4 uptake is presented for all adolescents 11-16 years combined because of low overall uptake. Td uptake is presented by single year of age from 11-16 years because 1) grouping distorted the patterns observed for single year of age and 2) Td recommendations did not parallel those of MCV4.

## Results

### MCV4 Uptake

The number of adolescents 11-16 years of age in the five MCOs totaled 623,889 (Table 1). Enrollment in the five MCOs ranged from about 13,000 to 298,000 and the two largest MCOs made up 90% of the study population. At the end of 2005, 64,272 vaccinations were administered and the overall coverage was 10% among adolescents 11-16 years of age (Table 1). Coverage was highest among 11-12 year olds (12.3%), followed by 14-15 year olds (10.5%), and lowest among the non-target, 13+16 year group (8.2%). The risk ratios for receiving MCV4 for ages 11-12 and 14-15 years compared to 13- plus 16-year-olds were 1.51 (95% Confidence Interval (CI): 1.48-1.54) and 1.29 (95% CI: 1.26-1.31), respectively. Of the 64,272 vaccinations, 38%, 35% and 27% were administered to 11-12, 14-15 and 13+16 year olds, respectively.

**Table 1:** MCV4 uptake during 2005 among 11-16 year old adolescents by MCO and age group

MCO	**Local or state MCV4 Legislation**^§^	**MCV4 shortage during 2005**^§^	Study Population	All adolescents Number (%) Vaccinated		Percent Vaccinated by Age Group		
						11-12	14-15	13+16 (Ref)
**A**	Yes^†^	No	19724	3680	(18.7)	33.7*	13.8*	9.6
**B**	No	No	31404	9518	(30.3)	31.9*	32.0*	27.0
**C**	No	Yes	13456	1832	(13.6)	15.2*	13.4	12.3
**D**	Unknown^‡^	Yes	261544	17934	(6.9)	6.4	7.7*	6.4
**E**	Unknown^‡^	Yes	297761	31308	(10.5)	13.9*	10.3*	7.5
**All MCOs**	--	--	623889	64272	(10.3)	12.3*	10.5*	8.2

MCV4 - Meningococcal conjugate vaccine

MCO - Managed care organization

^§^Based on self-report by principal investigator at each MCO

^†^MCO is located in a state with legislation requiring meningococcal vaccination for adolescents in residential schools and postsecondary institutions that provide housing for students.

^‡^MCO did not provide a response; however, the state(s) these MCOs are located in did(do) not have any meningococcal vaccination legislation[24].

*p < 0.001 when compared to 13+16 year group

The coverage by MCO for all adolescents combined ranged from 7% to 30%; the lowest coverage was in the two largest MCOs (D and E). Coverage for the 11-12 and 14-15 year groups ranged from 6% to 34% and 8% to 32%, respectively (Table 1). At each MCO, these two target age groups were more likely to be vaccinated than the non-target group (*p *< 0.001) with the exception of age group 14-15 at MCO C and 11-12 at MCO D. MCO C, D and E reported having some vaccine shortages. Of these three, MCO D recommended that given the shortage, providers selectively offer MCV4 only to patients at increased risk for meningococcal disease including college freshmen living in dormitories; adolescents 11-12 years and those starting high school were to be deferred to avoid exhausting MCV4 supplies.

**Figure 1:**
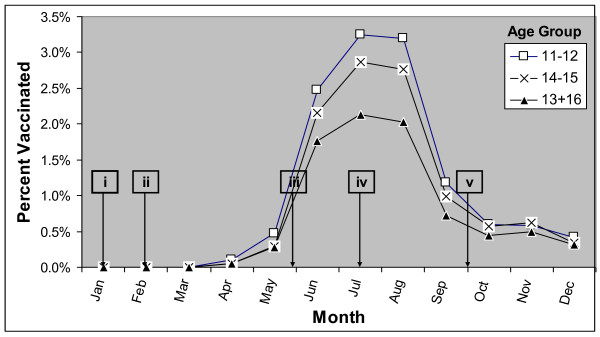
**Percent of 11-16 year-olds vaccinated with MCV4, for all 5 MCOs combined, 2005**. MCV4 - Meningococcal conjugate vaccine; MCO - Managed care organization. i - Jan 14, 2005 - Food and Drug Administration (FDA) licenses MCV4. ii - Feb 10, 2005 - Advisory Committee on Immunization Practices (ACIP) recommends MCV4. iii - May 30, 2005 - ACIP recommendations published (become official). iv - July 19, 2005 - MCV4 manufacturer announces limits on MCV4 orders. v - Sep 30, 2005 - FDA and Centers for Disease Control and Prevention announce an investigation of a potential link between MCV4 and Guillain-Barré Syndrome.

Figure 1 shows the percent of adolescents vaccinated by month and age group during 2005 for all 5 MCOs combined. Vaccine uptake began in March and started to increase in May, peaked in July, and declined until October, 2005, remaining level thereafter. Of the 64,272 total MCV4 vaccinations administered, 73% were administered during June, July and August (21%, 26% and 26%, respectively).

### MPSV4 Uptake

**Figure 2:**
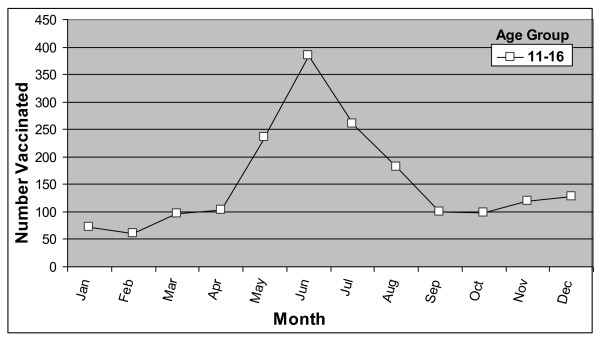
**Number of 11-16 year-olds vaccinated with MPSV4, for all 5 MCOs combined, 2003-2004**. MPSV4 - Meningococcal polysaccharide vaccine; MCO - Managed care organization.

During 2003-2004, a total of 1,837 MPSV4 vaccinations were administered to adolescents 11-16 years of age. Uptake for 11-16 year olds started to increase in May and peaked in June (Figure 2). Of the total doses, 45% were administered during June, July and August (21%, 14% and 10%, respectively).

### Td Uptake

**Figure 3:**
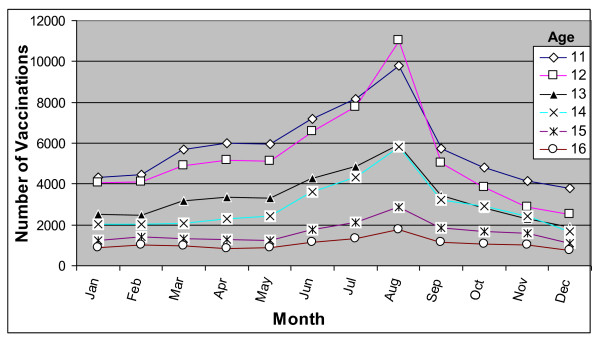
**Number of Td vaccinations administered to 11-16 year-olds, for all 5 MCOs combined, 2003-2004**. Td - Tetanus diphtheria vaccine; MCO - Managed care organization.

During 2003-2004, a total 240,480 Td vaccinations were administered to adolescents 11-16 years of age. The number of vaccinations administered decreased with age, ranging from 29% among 11 year olds to 5% in the 16 year olds (data not shown). Overall, adolescents 11 and 12, 13 and 14, and 15 and 16 years of age appear to have similar seasonal patterns with the number of vaccinations rising substantially from May to June and peaking in August (Figure 3). Additionally, 11-13 year olds also had a smaller increase in uptake starting in March. Of the total number of vaccinations, 10%, 12%, 15% were administered during June, July and August, respectively.

## Discussion

MCV4 coverage during the first ten months after its licensure (March-December 2005) was about 11% among targeted adolescents. Non-targeted adolescents (13 and 16 year olds) received a sizeable portion (27%) of the total administered doses. Of the five MCOs, the two largest reported vaccine shortages and had the lowest coverage. The surge in MCV4 uptake during the summer months coupled with the uptake among non-targeted adolescents likely contributed to the vaccine ordering limits and reported shortages. Seasonal uptake patterns of MPSV4 and Td (prior to MCV4 availability) show a similar increase in uptake during the summer, though the month of the peak uptake varied.

Prior to the availability of national coverage data,[14] most vaccination coverage information for adolescents came from cross-sectional or before-and-after studies designed to evaluate the impact of immunization requirements, voluntary school-based programs, and intervention designed to increase the delivery of clinical preventive services [15-20]. In general, these studies have revealed only limited uptake of newly recommended vaccines for adolescents before the implementation of interventions that were being studied. Other studies of vaccine uptake in adolescents have shown similarly modest uptake[8,21,22]. The first nationally representative physician-validated adolescent immunization survey (National Immunization Survey-Teen) found that MCV4 coverage among US adolescents 13-17 years was 12% during 2006[14]. Our results demonstrate that the VSD can be an important data source for timely evaluation of new or expanded vaccine recommendations given their use of a real-time surveillance system[12].

Vaccine uptake among adolescents appears to have a seasonal pattern. MCV4 uptake started increasing in May and peaked in July. Examination of MPSV4 and Td also shows increases in uptake during summer months. The earlier peak for MPSV4 suggests that different factors may play a role; however, given the small number of MPSV4 vaccinations (compared to Td and MCV4), it is difficult to hypothesize possible reasons. It is possible that MCV4 would have peaked in August (before the start of school), like Td, if not for the vaccine limits placed by the manufacturer (in July). Michigan's vaccine registry data showed a similar surge in MCV4 uptake during summer months suggesting that this seasonal increase is not limited to adolescents enrolled in managed care [21]. The GBS investigation was announced on September 30, 2005, and by then vaccine uptake had declined substantially. Although we cannot ascertain whether the decline between September and October was related to the GBS investigation, the impact of the investigation, if any, was likely minimal in this study population given the similar trend observed for Td.

Coverage was lower in the three MCOs reporting shortages (MCO C, D, and E) than in the other two. This included the two largest MCOs where implementation of a new vaccine recommendation is likely more challenging compared to smaller MCOs. Coverage differences within and between MCOs could have been affected by the nature of the shortage and/or the implementation plan at the MCO; we were unable to gather detailed information from all MCOs. The low coverage among the targeted adolescents in MCO D was likely related to their change in policy due to the shortage which instructed providers to defer vaccinating 11-12 and 14-5 year olds and preferentially vaccinate college freshman. School immunization laws have had a marked impact on both the incidence of vaccine preventable disease and immunization coverage in the United States. A limited number of studies suggest that much of the success of adolescent immunization programs in the United States is a direct result of these requirements[16,23]. During our study period, none of the MCOs were affected by statewide MCV4 legislation (Table 1)[24].

In February 2005, the ACIP carefully considered supply, anticipated demand and disease epidemiology when recommending routine use of MCV4 for adolescents 11-12 and 14-15 years of age[25]. At the same meeting, the manufacturer projected producing about 5 million doses in 2005[26] and the 2005 CDC Annual Biosurveillance data (CDC unpublished data) show that the manufacturer reported distributing about 3.1 million doses in 2005. The 2005 MCV4 mismatch between supply and demand and the reported shortages likely resulted from a combination of factors, including the increase in uptake over the summer, vaccination of non-targeted groups, and a supply shortfall possibly related to manufacturing capacity. Additionally, analysis of insurance claims data (presented by the MCV4 manufacturer at the June 2006 ACIP meeting) showed that from March-August 20, 2005, among adolescents, Menactra was administered more commonly to 18 year olds than other age groups [27]. This also likely contributed to the reported shortages. Uptake among 18 year olds could not be assessed in this study because not all MCOs collected data for this group. Based partly on our findings, a supply-demand imbalance was anticipated the following summer (2006) leading ACIP to recommend in May 2006 that MCV4 administration to 11-12 year olds be deferred; that supply limitation was resolved in November 2006[28,29]. In August 2007, ACIP changed their MCV4 recommendation to include routine vaccination of all adolescents 11-18 years partly to simplify provider decisions to vaccinate[30].

This study has some limitations. First, these results based on the VSD data may not be generalizable to uninsured adolescents and coverage during 2005 was likely lower in the overall US adolescent population; however, with standardized protocols, VSD data can be used to obtain timely vaccination coverage of recently and newly recommended vaccination. Second, we could not account for vaccinations that might have been administered outside the VSD MCO; however, considering the high price of MCV4 (list price in 2005 was $82), it is unlikely that many adolescents received this vaccine outside the plan. Third, this study is unable to account for the effect of any local events on seasonality of vaccination trends such as a case of meningococcal disease. Finally, we could have underestimated coverage among the target groups due to misclassification; some adolescents included in the 13+16 year-old, "non-target" age group, may in fact have been vaccinated according to recommendations if they were starting high school at those ages. However, the impact on our estimates is likely negligible given US school enrollment estimates.

## Conclusion

These data from the VSD confirm the anecdotal reports of providers that vaccinations increase during summer months. This seasonal trend is likely related to vaccination requirements for summer recreational activities, upcoming school requirements and convenience for parents. Parents need to recognize that regular preventive visits provide an opportunity for obtaining timely vaccinations. In addition, the comparatively high proportion of vaccine doses administered to adolescents outside of the target groups illustrates the challenges associated with implementing complex vaccination recommendations. These factors combined with the limitations in vaccine supply resulted in a failure to meet MCV4 demand during the summer of 2005, and should be considered in the future as new vaccines are recommended to avoid potentially preventable shortages. Avoiding supply disruptions is essential to a key aspect of increasing vaccination in adolescents: reduction of missed opportunities for vaccinations during all healthcare visits.

## Competing interests

The authors declare that they have no competing interests.

## Authors' contributions

SL: Participated in study conception and design, data analysis and drafting the manuscript. DF: Participated in study conception and design, data analysis and drafting the manuscript. EW: Participated in design, data collection and analysis and drafting the manuscript. PM: Participated in design and data analysis and drafting the manuscript. GL: Participated in design and data analysis and drafting the manuscript. FZ: Participated in design and data analysis and drafting the manuscript. RD: Participated in design and drafting the manuscript. All authors read and approved the final manuscript.

## Pre-publication history

The pre-publication history for this paper can be accessed here:

http://www.biomedcentral.com/1471-2334/9/175/prepub
